# The Cross-Cultural Validation of Neuropsychological Assessments and Their Clinical Applications in Cognitive Behavioral Therapy: A Scoping Analysis

**DOI:** 10.3390/ijerph21081110

**Published:** 2024-08-22

**Authors:** Evgenia Gkintoni, Georgios Nikolaou

**Affiliations:** Department of Educational Sciences and Social Work, University of Patras, 26504 Patras, Greece; gnikolaou@upatras.gr

**Keywords:** culturally adapted cognitive behavioral therapy, CA-CBT, depression, anxiety, PTSD, psychosis, randomized controlled trials, diverse populations, feasibility, acceptability

## Abstract

Objective: The present study explores the cross-cultural validation of neuropsychological assessments and their clinical applications in cognitive behavioral therapy (CBT), focusing on culturally adapted CBT (CA-CBT) across diverse populations and settings. Methods: Following the PRISMA guidelines, a comprehensive search was conducted in multiple academic databases, including PubMed, PsycINFO, Scopus, and Web of Science. Keywords related to cognitive behavioral therapy, cultural adaptation, and specific populations were used. The inclusion criteria encompassed randomized controlled trials (RCTs) and pilot studies that assessed CA-CBT for various mental health conditions. Results: The review included studies involving Chinese Americans, Latino caregivers, Syrian refugees, Jordanian children, Malaysian Muslims, Afghan refugees, Iraqi women, Japanese children and adolescents, and Tanzanian and Kenyan children. CA-CBT demonstrated significant effectiveness in reducing symptoms of depression, anxiety, PTSD, and psychosis. For instance, research has shown that CA-CBT is more effective than standard CBT in reducing depressive symptoms among Chinese Americans and in significantly lowering PTSD symptoms in Syrian refugee women. This method has been well-received and is feasible for use in diverse populations, such as Jordanian children and Afghan refugees. The long-term benefits are promising, with sustained improvements being reported in various studies. Additionally, digital and remote delivery methods have demonstrated potential for expanding the accessibility of CA-CBT. Conclusions: CA-CBT is a valuable and effective intervention for diverse cultural populations, significantly improving mental health outcomes. However, future research must address limitations such as small sample sizes, short follow-up periods, and variability in assessment tools. Future studies should include larger and more diverse sample sizes, longer follow-up periods, rigorous control groups, and comprehensive outcome measures to further validate and enhance the application of CA-CBT across different cultural contexts.

## 1. Introduction

Cognitive behavioral therapy (CBT) has been shown to be efficacious for the treatment of a wide array of psychological disorders. However, cross-culturally, there are issues with the validation of classical neuropsychological assessments, one of the tools used to detect cognitive deficits. Furthermore, the cross-cultural validation of neuropsychological assessments and their clinical applications in cognitive behavioral therapy is a crucial area of research aimed at ensuring the accuracy and effectiveness of cognitive assessments across diverse populations. As highlighted by a study [1], there is a recognized need to advance current approaches to neuropsychological assessments by incorporating modern psychometric theory and technological advancements to enhance the validity of assessments. This evolution is essential to address the challenges identified by the researchers of [2], who emphasized the necessity of developing and validating neuropsychological tests that are applicable across different cultural and linguistic backgrounds, especially in non-Western and low-educated populations. Another study [3] further underscored the importance of harmonizing cross-cultural neuropsychological data by developing culturally sensitive measures like the Multicultural Neuropsychological Scale and the Global Neuropsychological Assessment battery. Such initiatives are vital in addressing the urgent need for culturally appropriate assessments, as highlighted by a researcher [4] who emphasized the significance of considering language barriers, education levels, and cultural factors in cognitive assessments. The European Cross-Cultural Neuropsychological Test Battery and the Rowland Universal Dementia Assessment Scale were identified as well-validated tools across European countries, emphasizing the importance of culturally validated assessments. Moreover, studies [5,6], which were conducted in the European Union, advocated against race-based norms and stressed the importance of developing widely applicable cross-cultural tests that consider individual variability. These recommendations align with the efforts to enhance the cross-cultural validity of neuropsychological assessments, as supported by the European Consortium on Cross-Cultural Neuropsychology.

Traumatized regions often face significant psychosocial challenges, which can exacerbate the impact of trauma on affected populations. Individuals living in these areas are more likely to experience heightened levels of stress, anxiety, depression, and post-traumatic stress disorder (PTSD), as well as other mental health issues. These psychosocial problems are often compounded by the breakdown of social structures, the loss of support networks, and the ongoing uncertainty and instability that characterize such environments. The interplay between trauma and these psychosocial stressors can hinder recovery and exacerbate mental health symptoms, making it essential to address these issues within therapeutic interventions [7]. Culturally adapted cognitive behavioral therapy (CA-CBT) has shown promise in addressing these complex needs by incorporating cultural, linguistic, and contextual factors into treatment, thereby improving the relevance and effectiveness of interventions in traumatized areas.

It is important to note that the development of neuropsychological assessments began primarily in Western countries, and as a result, assessment tools are often founded on a mono-cultural concept. When these tools are applied overseas or to non-Western populations, they do not always function as designed. Few studies have explored the interactions between language, culture, and cognitive processes related to neuropsychological assessments [8,9,10,11,12,13,14,15,16,17,18,19,20,21,22,23]. The literature in this field can be divided into six general research perspectives, which are presented along with their findings:

(1) Studies involving the clinical assessment and diagnosis of cognitive functioning and cognitive change with respect to multiple languages and cultures, including both single and comparative case studies and more extensive sample size studies, for digital and non-digital neuropsychological assessments [14,15,16,24]. Theories related to cultural competence and neuropsychological assessments, such as those discussed in [25], support the need for culturally sensitive diagnostic practices. These models highlight how cultural factors influence cognitive assessment outcomes and stress the importance of adapting clinical practices to ensure validity across cultures.
(2) Validation studies for adapting tools to language-specific or culture-specific assessments [13,14,19,20,25,26]. The need for cross-cultural validation is supported by theoretical models of test adaptation and equivalence, such as those proposed in [27]. These models provide a framework for understanding how psychological assessments need to be adapted and validated to maintain their reliability and validity across different cultural contexts.
(3) Qualitative research on neuropsychological assessments, covering methods for cognitive function assessments, oral history or cultural memory for clinical condition evaluation, and intercultural research on the content and administration of cognitive tools in clinical research or everyday settings [11,15,16,17,28,29]. Theories from cultural psychology, such as those in [30,31], support the use of qualitative research to explore how culture shapes cognitive processes and behaviors, thus providing a theoretical basis for this category.
(4) The development and design of cognitive function assessments and experimental procedures considering indigenous needs for medical resources [18,32]. The theoretical underpinnings of this category can be found in models of test construction and cultural adaptation, such as those discussed in [33] in the context of adapting educational and psychological tests. These models emphasize the importance of considering cultural differences in the development of assessment tools, ensuring that they are culturally relevant and valid for the populations being tested.
(5) Theoretical papers and guidelines on the need to identify future directions and ethical considerations [21,34]. Theoretical discussions on ethics, such as those indicated in [35] on multicultural competence, provide a foundation for understanding the ethical challenges and considerations in applying neuropsychological assessments across different cultural groups.
(6) Practice session papers, including studies reporting the development of digital app-based tools [22,36]. The integration of technology into neuropsychological assessments is supported by theoretical models of digital and remote assessments, such as those discussed in [37] that explored the implications of using digital tools in psychological testing. Theories related to the digital divide and accessibility, such as those presented in [38], also provide a framework for understanding the challenges and opportunities associated with implementing digital assessments in diverse cultural contexts.

Moreover, the field of the cross-cultural validation of neuropsychological assessments is rapidly evolving with a focus on developing culturally sensitive and valid assessment tools for diverse populations. By incorporating the modern psychometric theory, technological advancements, and cultural considerations, researchers aim to ensure the accuracy and applicability of neuropsychological assessments in cognitive behavioral therapy across different cultural contexts.

This paper aims to clarify the relationship between plausibility concerning cognitive function assessment content and related language and cultural backgrounds. It reveals significant misalignments and considerations that should be accounted for when using assessment tools in a cross-linguistic or cross-cultural clinical setting. A further objective is to investigate how cross-cultural factors may interact with the choice of CBT strategies and, in particular, with the choice of a classical neuropsychological battery of tests or the need for unique neuropsychological tasks due to the emphasis on cultural perspective.

## 2. Literature Review

Several major debates on cross-cultural methodology have developed in recent years. These debates can be grouped into two points of view. The first perspective believes cultural groups should be examined separately using the traditional single-group approach. The second perspective emphasizes the equivalence of psychological measurements across cultural comparative studies. Researchers supporting the equivalence approach put more emphasis on measuring the psychological test. They claim it is very important to ensure equivalent measurements of the psychological test because it will affect group comparison, interpretation, and the direct application of the psychological test across different cultural groups [39,40,41].

A neuropsychological assessment is a focus of attention regarding differences in cognitive functioning across cultures. There are two main reasons for this. The first reason is driven by clinical efforts to address the unique needs of minority and indigenous language groups. Culturally sensitive clinical methods increase the likelihood that individuals from diverse backgrounds are accurately identified as potentially impaired in their cognitive abilities. The second reason is driven by research attempting to examine the effects of culture on neuropsychological functioning or whether different complex patterns of cognitive–behavioral functions are shared by individuals from the same cultural background [42,43].

In psychology, psychological tests are valuable methods to measure psychological constructs in people’s lives. However, when a psychological test developed in one culture is used with a different culture, it is necessary to conduct research to examine whether the psychological test is the same or equivalent across cultures. This process is called cross-cultural validation [44].

### 2.1. Importance of Cross-Cultural Validation

Different cultures develop different languages and communication patterns that may engage brain areas in different ways. The ways in which communication affects the way culture develops also involve animated components, including behaviors and gestures. They depend largely on and are influenced by the environment and vary over time as the environment changes. Brain changes derive from this bidirectional phenomenon, reflecting on cognitive skills that vary among specific groups, cultures, and countries. One example is language. Neuropsychological tests that assess cognitive, emotional, and social function, however, become relevant in the development of cognitive maps of the world when broader capabilities, knowledge, and strategies are required to negotiate social realities between different cultural communities. Cross-cultural validation studies are then inevitable to ensure data accuracy and fairness in such assessments. This procedure points out possible biases or discrepancies in the measurements. It thus provides an avenue for an adjustment to be made so that the measures are applicable and reliable across different cultural groups. The validation process also ensures that using such measures, there will be an accurate assessment of culturally diverse subjects’ cognitive and neuropsychological functions. This is crucial for ensuring that measures can be applied across and are effective in these different cultural groups.

Moreover, it helps deal with possible biases in assessments and promotes the accuracy of the results. This becomes of the essence, especially when dealing with a diverse population, since cultural differences may significantly affect the assessments’ competence [45,46,47].

The activity of neuropsychology is related to the brain–behavior link because significant actions and effects on the environment are in the cognitive domain. Since culture defines a particular society’s behaviors, norms, and values, it becomes essential to consider the potential effect of cultural variables when designing and administering neuropsychological tests. The understanding of the human mind as the result of ecological adaptiveness is a specific gift of the field of neuropsychology as it can validate neurobiological explanations. Neuropsychology, in fact, investigates the neural bases of several human behaviors sedimented on evolutionary foundations, giving reasons for why the brain is functional in so many different activities. This is particularly important when considering the diverse cultural contexts in which these assessments are used. It is essential to ensure that the assessments are culturally appropriate and relevant for the populations being served [48,49].

### 2.2. Cross-Cultural Considerations in Neuropsychological Assessments

The main purpose of a neuropsychological assessment is to identify a potential cognitive impairment and to quantify neuropsychological function across different domains with concern for different etiologies, among others. The key principle of a cross-cultural psychological assessment is to understand how the cultural background of the client influences their behavior, emotional state, and psychological needs and to adjust it to find the best possible way to evaluate mental status and functions. Commissioned by the Psychological Assessment of Racial and Ethnic Minority, for psychological assessments, although norm generation and cultural adaptation are desirable and, in some cases, essential, historically, it has been equally important to acknowledge and avoid negative consequences from racism and ethnocentrism, which are prevalent in the classification of neuropsychological function. Cross-cultural validation is crucial for ensuring the reliability and validity of neuropsychological assessments in diverse populations. It is important to consider cultural factors that may influence the interpretation of test results and the development of assessment tools. In particular, language barriers and differences in educational and occupational background can impact test performance. Therefore, it is crucial for neuropsychological assessments to be validated across different cultural and linguistic groups to ensure their accuracy and effectiveness in cognitive behavioral therapy [6,50,51].

Much attention has been given to the development of normative standards for neuropsychological tests that adequately correct for normal age-related changes in cognitive function. However, the contribution of cross-cultural influences is less understood. Even within the domains in which a particular test is most reliant, it may be possible that this tool is not equally sensitive across different populations. Currently, neuropsychological tests are well reviewed in terms of examining emotion-related and basic cognitive abilities and their underlying structures and implications for measuring therapeutic change [52]. Despite recent interest in this topic, limited research and recommendations are available for tests of neuropsychological function. Differences in the mean test performance between different cultural populations have been described across a range of neuropsychological and intelligence tests. These differences highlight the importance of culturally sensitive assessment techniques and the need for cross-cultural validation studies. In addition, understanding cultural factors that may impact performance on neuropsychological assessments is crucial for accurate diagnosis and effective treatment planning. It is important to consider cultural differences in cognitive processes and behaviors when interpreting neuropsychological test results [6,53,54,55,56].

### 2.3. Cognitive Behavioral Therapy: Principles and Applications

Cognitive behavioral therapy has been recognized as an evidence-based treatment for various psychiatric disorders. Developed from core elements such as behavioral activation and social learning theory, cognitive behavioral therapy is designed to be adjusted when applied in diverse cultural contexts. As cognitive behavioral therapy continues to exhibit the potential for treating broad populations, studies addressing cross-cultural issues in cognitive behavioral therapy are scarce, and the usage of cross-culturally validated neuropsychological assessments remains infrequent. This potentially limits the reliability of cognitive behavioral therapy provided to the respective individuals, especially when cognitive interventions for cognitive skills and functions are being proposed [57,58].

At present, the cross-cultural application of cognitive behavioral therapy is still limited, and evidence-based cognitive interventions for individuals of different cultural backgrounds remain scarce. There are concerns that the culturally specific themes and cognitions may have been neglected. While conducting a cultural formulation interview, clinicians need to attend to potential individual differences and symptoms such as somatic focusing expressions, interoceptive cognitive bias, emic explanations of basic emotions, stigma from mental health problems, interpretations and expressions of certain specific symptoms such as tinnitus, differing concerns about psychological symptoms, and differences between syndromal and idiom formulations. Although there is still no single dominant theme regarding how ethnic, cultural, and racial identity factors should be incorporated into the therapeutic process, future cognitive behavioral treatment implementation and case conceptualization should consider how treatments can be specifically tailored to the accumulation of knowledge of cultural cognitive factors or cultural focus points [59,60,61].

### 2.4. Fundamentals of Cognitive Behavioral Therapy

Cognitive behavioral therapy (CBT) is a psychotherapeutic approach based on the theories developed by Beck. This form of therapy has been established as one of the treatments of choice for various mental diseases. The emphasis of CBT is mainly on the individual’s present behavior and cognition and employs the collaboration of the patients and the therapists in the therapeutic process to rectify underlying distortions of thought and belief. CBT focuses on the modification of automatic thoughts, skilled practice in cognitive restructuring, and educational techniques to impart skills to patients to manage their emotional as well as physiological responses to environmental stimuli.

Fundamental to CBT is that not only can the behaviors, cognitions, and emotions be altered separately, but that modifications in these three aspects can affect the others. The behavioral component encompasses implementing experiments, either within or outside the structured session, to assess the soundness of hypotheses about automatic thoughts or underlying assumptions. Furthermore, it includes behavioral activation techniques to restore disrupted activities and effectively manage positive reinforcement. The service model delineates the necessary steps the patient should undertake to address the issue at hand effectively. The cognitive component emphasizes conscious thoughts about oneself, others, and the world and the maladaptive cognitive structures comprising thoughts, and it appraises patterns and schemas of core beliefs. The affective component uses various therapeutic methods designed to cope with emotions [62,63,64].

### 2.5. Integration with Neuropsychological Assessments

A neuropsychological assessment is an important tool for understanding memory, emotional, and personality characteristics associated with the occurrence of various psychiatric disorders, including trauma- and stressor-related disorders. Understanding the respective sensorium and developing a cross-cultural assessment are important strategies in CBT for trauma in multicultural populations. A cross-cultural approach is useful in cross-disciplinary areas, such as neuropsychology, which also encompasses clinical psychology. In this chapter, the essence and issues of neuropsychological assessments and “traumatic cognition” are introduced in relation to clinical psychology. Furthermore, CBT, ecological validity, and neuropsychological assessments in diverse groups from interpersonal, race, gender, and sexuality perspectives will be discussed through a three-tiered confrontation at the individual, physical, and social levels [65,66,67].

Neuropsychological assessments can be applied not only in studies of traumatic disorders, such as post-traumatic stress disorder (PTSD), but also in numerous other psychiatric problems such as neurodevelopmental disorders, eating disorders, addiction, and so on. For trauma assessments, “traumatic cognition”, which specifically occurs during a traumatic event, is important; the working factor in CBT is important for patients affected by trauma. Nevertheless, the lack of adequate preservation of essential cognitive processes, such as memory, presents challenges to effectively expressing thoughts related to traumatic events. To build an assessment approach that respects multiculturalism, it is necessary to grasp not only the ethological senses accumulated from the development environment but also the true feelings of individuals who survived the trauma, the will to live, the regret of survival, the benefits of recovery, and the personal feedback required for individual psychological control [68,69,70,71].

### 2.6. Scope and Objectives

This scoping analysis aims to provide a comprehensive evaluation of culturally adapted cognitive behavioral therapy (CA-CBT) across diverse populations and settings. This review encompasses randomized controlled trials (RCTs) and pilot studies, assessing the effectiveness, feasibility, and acceptability of CA-CBT for various mental health conditions such as depression, anxiety, PTSD, and psychosis. It includes interventions delivered through face-to-face, group sessions, telephone-based, and internet-delivered platforms.

It reviews the efficacy of CA-CBT in reducing symptoms of depression, anxiety, PTSD, and psychosis among different cultural groups. For instance, a study [72] further evaluated CA-CBT among the Chinese American population with depression, and another [73] evaluated the effectiveness of this approach in managing PTSD symptoms among Syrian refugee women. It also determines the applicability and acceptability of CA-CBT interventions within different cultural contexts; for instance, this variable was observed by studies like [74,75], among samples comprising Jordanian children and Afghan refugees, respectively.

It assesses the long-term effects and sustainability of CA-CBT interventions. The results at the follow-up from references [76,77] report on the durability of the treatment effects. For example, it reports precisely what cultural adaptation was made to enhance the effectiveness and acceptability of CBT, for instance, in studies like [72] involving Chinese Americans and [77] involving Malaysian Muslims. It compares the effectiveness of CA-CBT with standard CBT and other therapeutic interventions, as exemplified in [72], which compared CA-CBT with standard CBT for Chinese Americans, and [78], which compared CA-CBT with muscle relaxation for Latino women with PTSD.

This review evaluates the potential of digital and remote delivery methods for CA-CBT, with references [78,79] investigating the effectiveness of internet-delivered and web-based CA-CBT interventions. It addresses methodological issues, such as sample size, dropout rates, and assessment tools, as highlighted by studies like [72,80], to improve the reliability of the findings. Additionally, it provides insights and recommendations for future research on CA-CBT, identifying gaps in the current literature and suggesting directions to enhance the understanding and application of CA-CBT.

The review includes studies that describe the method used for cross-validation and not just present tests used in other countries or non-validated neuropsychological assessments. It covers books, book chapters, articles, reviews, dissertations, and theoretical proposals, in addition to descriptive and qualitative statistics studies published in Portuguese, Spanish, and English. The exclusion criteria include studies in the gray literature, including those focusing on other types of disorders, different methods to validate neuropsychological assessments, animal studies, and pediatric neuropsychology.

**Figure 1:**
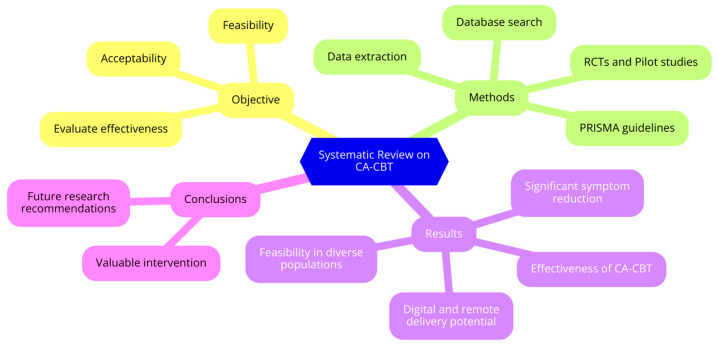
Flowchart of conceptual framework.

This review emphasizes the importance of using cultural filters in the application, interpretation, and validation of tests. By considering these cultural nuances, this review aims to present cognitive tests validated in other countries and their clinical applications in cognitive behavioral therapy (CBT). Specifically, by addressing these objectives, this review is focused on elaborating on CA-CBT’s efficacy, feasibility, and acceptability within different populations and settings. It thus becomes a similarly valid appraisal about the existing evidence and provides some leads on future research orientations (Figure 1).

### 2.7. Research Questions

- [RQ1] What are the long-term effects of CA-CBT on mental health outcomes across different cultural groups?
- [RQ2] What factors contribute to the success of CA-CBT, and how effective, feasible, and scalable are digital and remote CA-CBT platforms in diverse populations?
- [RQ3] What specific cultural adaptations are necessary for the effective implementation of CA-CBT in various cultural contexts?
- [RQ4] How does the effectiveness of CA-CBT compare to standard CBT and other therapeutic interventions in diverse populations?
- [RQ5] What are the key considerations in validating neuropsychological assessment tools for use in diverse cultural settings?
- [RQ6] How can neuropsychological assessments be adapted to better align with the cultural contexts of diverse populations receiving CBT?

These research questions aim to address the gaps and limitations identified in the current studies and guide future research to further validate and enhance the effectiveness, feasibility, and scalability of culturally adapted cognitive behavioral therapy across diverse populations and settings.

## 3. Materials and Methods

A scoping analysis was conducted following the methodology and framework commonly associated with a systematic review according to the guidelines of the Preferred Reporting Items for Systematic Reviews and Meta-Analyses. This combination allowed for a broad mapping of the literature (scoping analysis) while maintaining a systematic approach to data collection and synthesis. The objective was to identify the effectiveness, feasibility, and acceptability regarding culturally adjusted cognitive behavioral treatment across different populations and settings.

### 3.1. Search Strategy

Searches of the literature were conducted in major bibliographic databases—including PubMed, PsycINFO, Scopus, and Web of Science—using a combination of thesaurus and text words for the concepts of interest. The search strategy included a combination of keywords and Medical Subject Headings (MeSH) terms related to cognitive behavioral therapy, cultural adaptation, and specific populations. The following keywords were used:

- “Cognitive Behavioral Therapy” OR “CBT”;
- “Culturally Adapted” OR “Cultural Adaptation” OR “Culturally Sensitive”;
- “Depression” OR “Anxiety” OR “PTSD” OR “Psychosis”;
- “Chinese Americans” OR “Latino” OR “Syrian Refugees” OR “Jordanian” OR “Malaysian” OR “Afghan Refugees” OR “Iraqi Women” OR “Japanese Children” OR “Tanzanian” OR “Kenyan”;
- “Randomized Controlled Trial” OR “RCT” OR “Pilot Study”.

### 3.2. Inclusion and Exclusion Criteria

Studies were included if they evaluated the effectiveness, feasibility, or acceptability of culturally adapted cognitive behavioral therapy (CA-CBT), specifically focusing on randomized controlled trials (RCTs) and pilot studies involving diverse cultural populations. Only articles published in peer-reviewed journals and written in English were considered. Studies that did not involve a cultural adaptation of CBT, were non-randomized, case reports, reviews, or not published in English were excluded. Additionally, studies without sufficient data on outcomes were also excluded. Only one case report was included in the table to illustrate the diversity of CA-CBT applications and to emphasize the importance of cultural and religious considerations in the effective implementation of CBT. It serves as an example of how CA-CBT can be tailored to meet the unique needs of individuals from specific cultural and religious backgrounds, a theme that is central to the objectives of the present scoping analysis.

Data were extracted by capturing study characteristics (author, year, country, sample size, and population), intervention details (type of CA-CBT, duration, and delivery method), outcome measures (depression, anxiety, PTSD, psychosis, and quality of life), main findings (effectiveness, feasibility, and acceptability), and follow-up duration.

A narrative synthesis summarized the findings from the included studies. The effectiveness of CA-CBT was evaluated based on the reported outcomes, including symptom reduction and quality of life improvements. Feasibility and acceptability were assessed based on dropout rates, adherence, and participant feedback.

**Figure 2:**
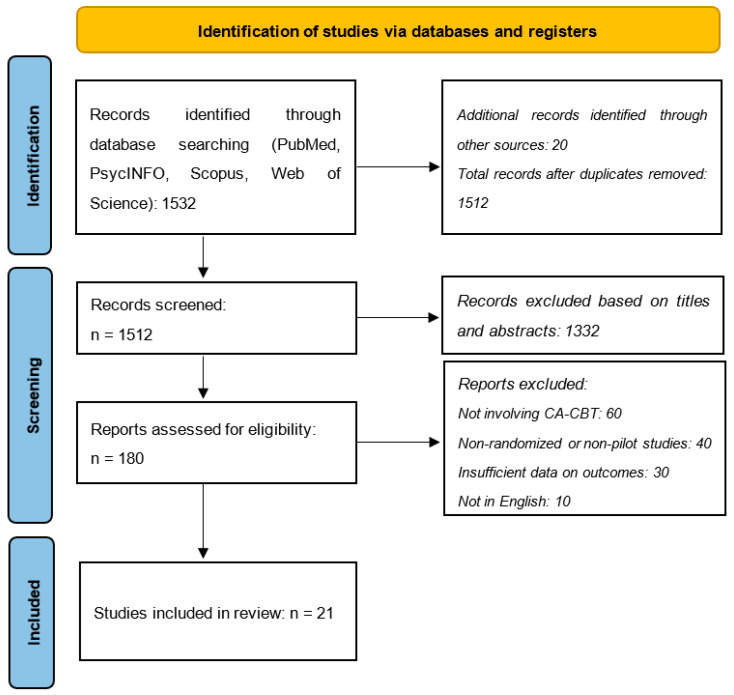
Flowchart of PRISMA methodology.

The study selection process was illustrated using a PRISMA flow diagram (Figure 2), detailing the stages of identification (number of records identified through database searching), screening (number of records screened and excluded based on titles and abstracts), eligibility (number of full-text articles assessed for eligibility and excluded with reasons), and inclusion (number of studies included in the final synthesis) [81]. Additional methodological details are available in the File S1 (Supplementary Material). Table 1 summarizes key details from the 21 studies included in the scoping analysis, including the study year, country, cultural context, neuropsychological assessment tools, sample sizes, and validation methods. It highlights the primary findings on the effectiveness and feasibility of culturally adapted cognitive-behavioral therapy (CA-CBT) across diverse populations, along with any identified gaps and limitations in the research (Table 1).

## 4. Results

### 4.1. Effectiveness of Culturally Adapted CBT

The effectiveness of CA-CBT refers to the overall success of the therapy in achieving its intended outcomes within specific cultural contexts. This includes not only symptom reduction but also the acceptability, feasibility, and cultural relevance of the therapy. Effectiveness encompasses the degree to which CA-CBT is well received by the target population, the extent to which it can be successfully implemented in different cultural settings, and its ability to engage participants in the therapeutic process. It also considers long-term outcomes, such as sustained improvements in mental health and overall well-being.

On the other hand, symptom reduction specifically refers to the measurable decrease in the severity of psychological symptoms, such as depression, anxiety, PTSD, or psychosis, as a direct result of the therapeutic intervention. Symptom reduction is a crucial component of effectiveness but does not capture the full scope of what makes CA-CBT successful. Symptom reduction can be quantified through various clinical scales and assessments used within the studies, which provide direct evidence of the therapy’s impact on mental health conditions. Additionally, while symptom reduction is an essential indicator of the effectiveness of CA-CBT, the broader concept of effectiveness also includes factors such as cultural adaptation, patient engagement, feasibility, and the sustainability of the therapy’s benefits.

Multiple studies have demonstrated the effectiveness of culturally adapted cognitive behavioral therapy (CA-CBT) across various populations. A study [72] found that CA-CBT led to a greater overall decrease in depressive symptoms among Chinese Americans compared to standard CBT, although both groups showed significant symptom reduction. Similarly, another study [73] reported that CA-CBT significantly reduced PTSD symptoms and anxious-depressive distress among Syrian refugee women, with large effect sizes for PTSD (HTQ d = 1.17) and nearly medium effect sizes for anxious-depressive distress (HSCL d = 0.40).

In the context of psychosis, two studies [83,89] found that CA-CBT significantly improved positive and negative symptoms, overall psychotic symptoms, and insights among patients in Pakistan. These improvements were statistically significant across various measures, including PANSS and PSYRATS.

For Latino populations, a researcher [82] demonstrated that a culturally sensitive CBT group intervention for Alzheimer’s caregivers led to lower neuropsychiatric symptoms in their relatives, less caregiver distress, greater self-efficacy, and fewer depressive symptoms over time compared to a psychoeducational control group. Another researcher [84] also found that CA-CBT significantly reduced PTSD symptoms in Latino women with treatment-resistant PTSD with very large effect sizes (Cohen’s d = 2.6).

### 4.2. Feasibility and Acceptability

The feasibility and acceptability of CA-CBT have been supported across different cultural contexts. A researcher [74] found that TF-CBT was feasible and well accepted among Jordanian children with abuse histories, with significant post-treatment improvements in PTSD and depression symptoms. Similarly, another researcher [75] reported that CA-CBT+ was feasible and acceptable among Afghan refugees, with large improvements in general psychopathological distress and quality of life and a low dropout rate.

A study [80] demonstrated that telephone-based CBT was feasible and acceptable for Latino patients in rural areas, with significant improvements in depression outcomes and high patient satisfaction. A researcher [77] also found that a culturally and religiously adapted CBT for a Malaysian Muslim with panic disorder was effective, leading to significant reductions in anxiety and panic attack symptoms.

Several studies have shown that the benefits of CA-CBT are sustained over time. A study [76] found that significant reductions in PTSD, depression, anxiety, and stress symptoms among Iraqi women were maintained at a 1-month follow-up. Additionally, another researcher [75] reported that improvements in general psychopathological distress and quality of life among Afghan refugees were sustained at a 1-year follow-up.

### 4.3. Digital and Remote Interventions

The potential of digital and remote interventions has also been explored. A researcher [78] described the cultural adaptation and implementation of an internet-delivered CBT program for depression in Colombia, aiming to establish a methodology for culturally adapting such interventions. Also, another researcher [79] found that adding a culturally adapted web-based CBT to standard treatment significantly improved substance use outcomes among Spanish-speaking individuals, demonstrating the potential of digital interventions to address health disparities.

### 4.4. Specific Populations and Settings

The studies also highlight the importance of tailoring CA-CBT to specific populations and settings. Also, a study [85] found that a bidirectional cultural adaptation of CBT for children and adolescents with anxiety disorders in Japan led to significant improvements in anxiety and depression symptoms. A researcher [86] explored the delivery of TF-CBT by lay counselors in Tanzania and Kenya, emphasizing the importance of cultural responsiveness and the value of TF-CBT for improving child mental health in low- and middle-income countries.

The provided papers collectively explore the effectiveness and feasibility of culturally adapted cognitive behavioral therapy (CA-CBT) across various populations and settings, highlighting the importance of cultural sensitivity in mental health interventions.

A researcher [72] conducted a randomized controlled trial (RCT) comparing standard CBT and CA-CBT for Chinese Americans with major depression. The study found that while both treatments significantly reduced depressive symptoms, the CA-CBT participants showed a greater overall decrease in symptoms. However, the majority of participants did not reach remission, suggesting that more intensive and longer treatments may be necessary for severe depression.

Furthermore, in another study, a researcher [82] tested the effectiveness of a culturally sensitive CBT group intervention, Circulo de Cuidado, for Latino Alzheimer’s caregivers. The study revealed that CBT participants reported lower neuropsychiatric symptoms in their relatives, less caregiver distress, greater self-efficacy, and fewer depressive symptoms over time compared to a psychoeducational control group.

Two other studies [83,89] evaluated the use of CA-CBT for psychosis in Pakistan. Both studies demonstrated significant improvements in positive and negative symptoms, overall psychotic symptoms, and insights among participants receiving CA-CBT compared to treatment as usual (TAU). These findings support the efficacy of CA-CBT in reducing psychotic symptoms and improving insights in low-income settings.

Additionally, another researcher [73] assessed the use of CA-CBT for Syrian refugee women in Turkey, finding that the intervention significantly reduced PTSD symptoms and anxious-depressive distress. The study also reported low dropout rates and no adverse events, indicating the treatment’s feasibility and acceptability.

Moreover, a study [74] examined the feasibility and acceptability of trauma-focused CBT (TF-CBT) for children with abuse histories in Jordan. The study found significant post-treatment improvements in PTSD and depression symptoms with sustained treatment gains at a 4-month follow-up.

Also, a researcher [77] implemented a culturally and religiously adapted CBT for a Malaysian Muslim with panic disorder and agoraphobia. The intervention led to significant reductions in anxiety and panic attack symptoms, demonstrating the effectiveness of incorporating cultural and religious elements into CBT.

A study [80] tested telephone-based CBT for Latino patients in rural areas, finding that the intervention significantly improved depression outcomes compared to enhanced usual care. The study highlighted the potential of telephone-based CBT to enhance access to psychotherapy in underserved populations.

In another study, the researchers [88] explored the relationship between brain-behavioral adaptability and the response to CBT for emotional disorders. The study found that individuals with greater brain-behavioral adaptability were more likely to respond to the treatment and show improvements in depressive symptoms.

## 5. Discussion

The results highlight that culturally adjusted CBT works feasibly and effectively and is well accepted across different populations and settings [90]. CA-CBT showed remarkable benefits for the diminution in symptoms of depression, anxiety disorder, post-traumatic stress disorder, and psychosis, hence proving its effectiveness in various cultural contexts [91]. These findings thus reflect a huge potential for CA-CBT in effectively addressing the psychiatric mental health needs of culturally diverse populations [92,93].

These studies have continually demonstrated the efficiency of CA-C CBT in mental health outcomes. For example, compared to standard CBT, CA-CBT was more effective in reducing depressive symptoms among Chinese Americans and significantly decreased PTSD symptoms in Syrian refugee women. The other discussed results of CA-CBT also include improvements in psychotic symptoms in Pakistani patients and promotions of neuropsychiatric symptoms with reductions in caregiver distress in Latino Alzheimer’s caregivers [94,95,96,97]. It is, however, the strong reduction in symptoms of PTSD and anxious-depressive distress in Syrian refugee women—accompanied by large effects for PTSD and close to medium effect sizes for anxious-depressive distress—that ultimately proves the versatility and efficacy of CA-CBT. These findings underline CA-CBT’s ability to tailor psychological requirements from different cultural groups of clients and therefore enhance appropriateness and impact.

Across the studies, feasibility and acceptability for CA-CBT were supported, suggesting that culturally sensitive adaptations of CBT are well tolerated by participants. For example, CA-CBT was feasible and acceptable among Jordanian children with histories of abuse, Afghan refugees, and Malaysian Muslims. Internet-delivered and telephone-based CBT also showed some promise in digital and remote delivery methods to improve access to CA-CBT in general, particularly within under-resourced and rural areas. These models were effective and acceptable, so they will probably contribute to overcoming barriers to access mental health care.

Within the context of psychosis, CA-CBT significantly improved positive and negative symptoms, overall psychotic symptoms, and insights among patients with psychosis in Pakistan. These were statistically significant across a range of measures, such as the PANSS and PSYRATS, which really illustrate the potential of CA-CBT in addressing severe mental health conditions within culturally diverse settings. In the case of Latino populations, a culturally sensitive CBT group intervention for Alzheimer’s caregivers evoked fewer neuropsychiatric symptoms in their relatives; therefore, it caused fewer distressed feelings in caregivers, enhanced self-efficacy, and resulted in a lesser degree of depression over time compared to a psychoeducational control group.

The review also pointed out that CA-CBT needed to be tailored for specific populations and settings. Examples include the bidirectional cultural adaptation of CBT for anxiety disorders in children and adolescents in Japan, resulting in high improvements in anxiety and depression symptoms, and TF-CBT delivery by lay counselors in Tanzania and Kenya, underscoring cultural responsiveness and the value of TF-CBT in improving child mental health in low- and middle-income countries.

The studies also pointed to the fact that the benefits of CA-CBT are retained over time. Iraqi women maintained a significant reduction in the symptoms of PTSD, depression, anxiety, and stress at a 1-month follow-up. It was found that Afghan refugees still had reductions in general psychopathological distress and improvements in quality of life at a 1-year follow-up, indicating how effective CA-CBT interventions can be in the long run.

On the other hand, digital and remote interventions have held an exceptionally special place due to their ability to make mental health services more accessible and inclusive. In the last phase, the cultural adaptation and implementation of the internet-delivered CBT program for self-help in depression in Colombia provided a first approach to the methodology for the cultural adaptation of this kind of intervention. Enhancement with culturally adapted, web-based CBT resulted in a great improvement in substance use outcomes among Spanish-speaking patients, thus pointing to the role of digital interventions in reducing health disparities [98].

Moreover, CA-CBT represents at least a moderately useful and effective intervention for a diverse range of cultural populations with the ability to produce significant improvements in mental health outcomes. One important mechanism by which this increased relevance and efficacy of the treatment method may be achieved is through the degree to which CBT can be adapted into these different populations to address relevant cultural and contextual needs, thereby making it an essential tool in global mental health care. The persistence of the benefits, feasibility, and acceptability of CA-CBT in different settings and modes of delivery represent the key potential magnitude of dissemination that can be realized during wide-ranging implementation to achieve better mental health outcomes for culturally diverse populations worldwide.

To sum up, it is important to mention that in the studies reviewed, several steps were taken to ensure that the culturally adapted cognitive behavioral therapy (CA-CBT) was both culturally competent and effective for the diverse populations involved. Language and communication were carefully considered, with adaptations made not just to translate the therapy but to make it culturally relevant. This involved using idiomatic expressions and culturally specific references, ensuring that the content was relatable and understandable for participants from different cultural backgrounds.

The therapy, in these respects, is embedded with the cultural beliefs and practices of the participants; they are imbibed into the cognitive and behavioral components of the therapy. For example, beliefs in religions were embedded in the cognitive restructuring of the Syrian refugees, while family dynamics were embedded in the treatment for the Chinese Americans. This made it congruent with the worldviews and lived experiences of the patients. The intervention tactics and content were also adapted to include culture-specific sources of stress and coping strategies. For Latino caregivers, the intervention focused on unique cultural experiences and sources of stress with coping strategies that were in line with Latino cultural values, such as familism, respect, and personal relationships [72,73,82].

Finally, the therapists had training sessions in cultural competency to assist them when working with participants from diverse backgrounds. These sessions helped prepare the therapists to provide this therapy in a respectful way but also in a way that was attuned to the cultural nuances of the individual being treated. This further comprises the overall effectiveness of the CA-CBT interventions. All of these steps together ensured that the reduction in symptoms, shown by the therapy’s effectiveness, was culturally relevant and well received by the participants.

### 5.1. Limitations

The scoping analysis presents valuable insights into the effectiveness and feasibility of culturally adapted cognitive behavioral therapy (CA-CBT) across diverse populations. However, there are several limitations that must be heeded with respect to interpreting the results. This scoping review was undoubtedly limited by the availability of studies meeting the selected criteria. This greatly limits the capacity for generalization to be applied to broad conclusions made from the review. This demands, with respect to very careful generalization, that these findings could be noted for the purpose of other cultural contexts because this small sample of studies might not reflect all of the diversity and complexity of the existing CA-CBT applications worldwide. Considering that only a limited number of studies were reviewed, one should refrain from overgeneralizing the findings. In fact, the conclusions are context-bound and must be interpreted with caution—particularly in terms of generalization for a different cultural or clinical setting.

Specifically, small sample sizes in many studies imposed a limitation that affects generalizability. For instance, in [73], there were only 23 participating Syrian refugee women, a small size that undermines generalization to a larger population at base. The same applies to [84], which had only 24 Latina women who had treatment-resistant PTSD. These findings cannot be generalized to the larger population of Latinos who have been affected by PTSD.

Furthermore, the follow-up periods in most studies were relatively short, allowing for little insight into the long-term effectiveness of CA-CBT. In particular, [76] reported the follow-up results of the treatment at only 1-month post-treatment, which may have missed sustained treatment effects in the longer time frame. Most participants had not reached remission at the end of the 12-week treatment period, as [72] commented, indicating that a longer follow-up is required to capture the full effect of the interventions.

Some of the studies, however, did not have appropriate control groups to compare the results with, so their findings cannot be attributed purely to the CA-CBT intervention. For example, ref. [77] had a single-case design and thus was without a control group; therefore, definitive conclusions about the effectiveness of the adapted CBT could not be drawn.

These cultural adaptations of the studies were population- and setting-specific, which might undermine the generalizability of the results to other cultural contexts. For instance, the cultural adaptation used for Chinese Americans in the study cannot be directly applied to other Asian American sub-groups or different ethnic populations. Likewise, the adjustments made for Jordanian children in [74] might not be generalizable to children in other Middle Eastern countries.

Some other studies had some methodological limitations, which might affect the validity of the findings; for example, the authors of [80] mentioned a small sample size and selection bias since the subjects were taken from only one rural medical center. Additionally, the study presented in [78], on Internet-delivered CBT, was not long-term in nature; therefore, this would not allow for the durability of the effects to be measured.

Some studies have reported high dropout rates and problems with treatment adherence, which may affect the reliability of the results. In this regard, the study reports dropout rates nearing significance: 26% in the CBT group compared to 7% in the CA-CBT group. This means that this type of dropout can create some bias and affect the interpretation of the results in relation to the effectiveness of the treatment.

The methodologies of different studies regarding measurement assessment tools can make comparison difficult to interpret directly. For example, the Harvard Trauma Questionnaire and Hopkins Symptom Checklist-25 were applied in [73], but the investigators used the Hamilton Depression Rating Scale in [72]. A cross-study comparison can be complicated due to differences in the results because variations in assessment tools for the same measurement can result in variations in the outcomes.

Some studies were specifically focused on certain elements of mental health, and it is possible that they do not represent the full range of benefits or limitations of CA-CBT. For instance, while a study was conducted to assess neuropsychiatric symptoms and caregiver distress [82], it did not examine other potential benefits such as quality of life or social functioning. 

In other words, while these studies offer valuable insights into the effectiveness and feasibility of CA-CBT, they are constrained by such limitations as small sample sizes, short follow-up periods, a lack of control groups, cultural and contextual variability, methodological constraints, dropout rates, variability in the measurement tools, and the extent and scope of interventions. Therefore, future research should try to surmount these limitations in order to arrive at more substantial and generalizable results.

### 5.2. Future Research

Future studies need to include larger and more culturally diverse samples to improve generalization. As sample sizes increase and incorporate different cultural groups, there will be a more substantial endorsement of the cross-population utility of CA-CBT. Longitudinal follow-up times are important in examining the effect of treatment maintenance. Long-term follow-up assessments need to be incorporated in future research, and it is important to examine whether the benefits of CA-CBT are sustained in the long term and/or if long-term effects and relapses occur.

Including appropriate control groups and maintaining rigorous randomization methods will be very important to establish the efficacy of CA-CBT. Future studies are to include well-described control conditions in a bid to strengthen causation inferences. Future research should also explore cross-cultural comparisons to understand how such cultural adaptations can result in CA-CBT interventions in different cultural contexts. The cultural adaptations implemented in studies like [72,74] are relative to their respective populations. Such cross-cultural comparisons will help advance our greater understanding of the universal and culture-specific elements of CA-CBT. Future studies need to use a wider array of measures to assess the full impact of CA-CBT. In general standards that extend to social skills and other relevant areas, outcomes should be measured. Measures of general quality of life, social functioning, and other relevant areas are required in this respect in future studies. In the future, it will also be necessary to evaluate the effectiveness, feasibility, and scalability in different cultural settings of CA-CBT delivered via the internet/telephone. This can help overcome barriers to access and extend service reach in mental health. The determination of the factors that contribute to dropout and the creation of adherence enhancement strategies will, in turn, improve the reliability and effectiveness of CA-CBT interventions. In fact, future studies should promote methodological improvements in rigor by attending to standard indicators and sources of measurement across studies to enable cross-study comparisons and increase the validity of the findings. Exploring the integration of CA-CBT with other therapeutic approaches and community resources might provide a more holistic treatment framework. Further research is required to investigate the benefits of integrating CA-CBT with other evidence-based interventions. As such, lay counselors can be capacitated on CA-CBT to be able to bridge this gap in mental health treatment. More research is needed to evaluate its impact and sustainability.

In conclusion, future research should focus on larger and more diverse sample sizes, long-term follow-ups, rigorous control groups, cross-cultural comparisons, comprehensive outcome measures, digital and remote interventions, addressing dropout rates, methodological rigor, integration with other interventions, and task-sharing approaches. These efforts will help to further validate and enhance the effectiveness, feasibility, and scalability of culturally adapted cognitive behavioral therapy across diverse populations and settings.

## 6. Conclusions

This scoping analysis underscores the significant efficacy and utility of culturally adapted cognitive behavioral therapy (CA-CBT) in improving mental health outcomes across diverse populations. The evidence consistently demonstrates that CA-CBT is effective in reducing symptoms of depression, anxiety, PTSD, and psychosis among various cultural groups. The culturally tailored interventions not only enhance the therapeutic impact but also improve the feasibility and acceptability of CBT in non-Western contexts.

The key findings indicate that CA-CBT leads to greater overall reductions in depressive and PTSD symptoms compared to standard CBT. In summary, CA-CBT represents a valuable and effective intervention for diverse cultural populations, with significant potential to improve mental health outcomes globally. Addressing the identified limitations through further research will help to confirm the effectiveness, feasibility, and scalability of CA-CBT, ultimately contributing to more inclusive and culturally sensitive mental health care practices worldwide.

## Figures and Tables

**Table 1:** Main results of scoping analysis (N = 21).

Study Year	Country/Culture	Neuropsychological Assessment	Sample Size	Validation Methodology	Key Findings	Application in CBT	Gaps/Limitations
Hwang, 2015 [72]	USA/Chinese Americans	Hamilton Depression Rating Scale	50	RCT	CA-CBT led to greater overall decrease in depressive symptoms	Enhanced effectiveness of CBT through cultural adaptation	Small sample size, short follow-up period, high initial severity of depression
Gonyea, 2016 [82]	USA/Latino	Neuropsychiatric Inventory, CES-D	67	RCT	Lower neuropsychiatric symptoms, less caregiver distress, greater self-efficacy	Culturally sensitive group CBT for caregivers	Limited to caregivers, short follow-up period
Habib, 2014 [83]	Pakistan	PANSS, PSYRATS, Insight Scale	42	RCT	Significant improvement in positive, negative, and overall psychotic symptoms	CA-CBT effective for psychosis in low-income settings	Small sample size, short follow-up period
Eskici, 2021 [73]	Turkish/Syrian Refugees	Harvard Trauma Questionnaire, Hopkins Symptom Checklist-25	23	RCT	Significant reduction in PTSD and anxious-depressive distress	CA-CBT effective for PTSD in refugee populations	Small sample size, short follow-up period
Damra, 2014 [74]	Jordan	PTSD and Depression Symptomatology	18	RCT	Significant post-treatment improvements in PTSD and depression symptoms	Feasibility and acceptability of TF-CBT in Jordanian culture	Small sample size, short follow-up period
Subhas, 2021 [77]	Malaysia/Muslim	Beck Anxiety Inventory	1	Single-case study	Significant reduction in anxiety and panic attack symptoms	Culturally and religiously adapted CBT for panic disorder	Single-case study, no control group
Dwight-Johnson, 2011 [84]	USA/Latino	Hopkins Symptom Checklist, Patient Health Questionnaire-9	101	RCT	Significant improvement in depression outcomes	Feasibility and acceptability of telephone-based CBT	Small sample size, potential selection bias
Zemestani, 2022 [76]	Iraq	PTSD Symptom Severity, Depression, Anxiety, Stress	48	RCT	Significant reductions in PTSD, depression, anxiety, and stress symptoms	CA-TF-CBT effective for war-related PTSD	Short follow-up period
Kananian, 2020 [75]	German/Afghan Refugees	General Health Questionnaire, PTSD Checklist, Patient Health Questionnaire	24	RCT	Large improvements in general psychopathological distress and quality of life	CA-CBT+ effective for refugees, low dropout rate	Small sample size, short follow-up period
Hinton, 2011 [78]	USA/Latino	PTSD Symptom Scale, Anxiety Measures	24	RCT	Significant reduction in PTSD symptoms, large effect sizes	CA-CBT effective for treatment-resistant PTSD	Small sample size, short follow-up period
Ishikawa, 2019 [85]	Japan	Diagnostic Interview, Self-Report Measures of Anxiety and Depression	51	RCT	Significant improvements in anxiety and depression symptoms	Bidirectional cultural adaptation of CBT for children and adolescents	Short follow-up period
Salamanca-Sanabria, 2018 [79]	Colombia	Patient Health Questionnaire-9	Not specified	RCT	Protocol for assessing efficacy of internet-delivered CBT	Methodology for culturally adapting internet-delivered interventions	No results reported, protocol study
Paris, 2018 [80]	USA/Spanish-speaking	Substance Use Frequency	92	RCT	Significant improvement in substance use outcomes	Web-based CBT effective for substance use disorders	Short follow-up period
Woods-Jaeger, 2017 [86]	Tanzania/Kenya	Qualitative Interviews	12	Qualitative Study	Importance of cultural responsiveness, value of TF-CBT for child mental health	Task-sharing approach for delivering TF-CBT	Small sample size, qualitative study
Palic, 2009 [87]	Denmark/Refugees	Harvard Trauma Questionnaire, Trauma Symptom Checklist-23	26	Observational Study	Small to medium effect sizes on most outcome measures	Multidisciplinary treatment including CBT for traumatized refugees	Small sample size, observational study
Stange, 2017 [88]	USA	Symptom Checklist-90-Revised, Depression Anxiety Stress Scales	39	RCT	Greater brain-behavioral adaptability predicted better treatment response	Brain-behavioral adaptability as a predictor of CBT response	Small sample size, short follow-up period
Husain, 2017 [89]	Pakistan	PANSS, PSYRATS, Schedule for Assessment of Insight	36	RCT	Significant improvements in positive and negative symptoms and overall psychotic symptoms	Feasibility and acceptability of CA-CBT for psychosis	Small sample size, short follow-up period

## Supplementary Materials

The following supporting information can be downloaded at: https://www.mdpi.com/article/10.3390/ijerph21081110/s1, File S1: PRISMA 2020 Checklist.
